# Genetic Diversity and Population Structure Analysis of Hoary Basil (*Ocimum americanum* L.) Germplasm From Burkina Faso Using Simple Sequence Repeat (SSR) Markers

**DOI:** 10.1155/tswj/5778077

**Published:** 2026-04-03

**Authors:** Hervé Kaboré, Monique Soro, Ezechiel Bionimian Tibiri, Fidèle Tiendrebéogo, Kiswendsida Romaric Nanema

**Affiliations:** ^1^ Department of Plant Biology and Plant Physiology, Joseph Ki-Zerbo University, Ouagadougou, Burkina Faso; ^2^ Department of Plant Production, Institute of Environment and Agricultural Research (INERA), Ouagadougou, Burkina Faso

**Keywords:** Burkina Faso, genetic diversity, *lamiaceae*, *Ocimum americanum*

## Abstract

*Ocimum americanum* is a wild, aromatic species belonging to the *Lamiaceae* family. It holds significant socioeconomic importance as it is widely utilized in agriculture, medicine, and food by the local population in Burkina Faso. Previous works revealed that *O. americanum* is one of the candidate species for domestication in Burkina Faso. Such a perspective requires a good understanding of its genetic diversity. This study was carried out to assess the genetic diversity of *O. americanum* based on simple sequence repeat (SSR) markers, also known as microsatellites. To achieve this goal, 79 accessions of *O. americanum* collected in three phytogeographic sectors (sub‐Sahelian, North Sudanian, and South Sudanian) were genotyped using 13 SSR markers. Nine SSR markers out of 13 revealed polymorphism within the accessions with PIC values ranging between 0.047 and 0.443. In all, 36 alleles were identified and the expected heterozygosity ranged from 0.047 to 0.449. Significant differences were observed between the accessions based on the phytogeographic sectors of origin. The analysis of the organization of the genetic diversity using the STRUCTURE approach led to two genetic groups, while the discriminant analysis of principal components (DAPC) revealed six groups. This study is the first report on the genetic diversity of *O. americanum* in Burkina Faso. The results of this study could help in developing a strategy for the sustainable management and utilization of the genetic resources of hoary basil.

## 1. Introduction

Hoary basil (*Ocimum americanum* L.), also known as African basil or lemon basil, is an important aromatic and medicinal herb widely distributed in tropical and subtropical regions [1, 2]. In Burkina Faso, hoary basil holds significant cultural, culinary, and medicinal value, making it a subject of interest for both traditional and scientific communities [3, 4]. It was identified as one of the candidate species for domestication in Burkina Faso [4]. From a taxonomic and cytogenetic perspective, *O. americanum* has attracted scientific attention due to its chromosomal characteristics.

*O. americanum* is classified as a tetraploid species with 2n = 4x = 48 chromosomes [5]. It stands out as one of the valuable wild species within the *Ocimum* genus.

Beyond its cytogenetic features, the species is also distinguished by well‐defined morphological traits, which are essential for accurate germplasm characterization and identification of accessions. This species is a semiwoody aromatic herb, reaching approximately 50 cm in height. It features a quadrangular, erect, and branched stem, densely pubescent. The leaves are simple, opposite, ovate to elliptical, measuring up to 2.5 cm in length, sparsely pubescent, with a glandular base that is dentate and attenuate, and serrate margins distally [6, 7]. The flowers are white or purplish‐pink, showy, with persistent subtending bracts.

Given its ecological, cultural, and agronomic importance, understanding the genetic variability of this species becomes essential. Assessing the genetic diversity of hoary basil germplasm in Burkina Faso is crucial for informing conservation strategies, optimizing utilization, and guiding breeding programs. This knowledge is vital for preserving the plant’s role in agriculture and medicine in Burkina Faso [8]. Genetic diversity plays a crucial role in the adaptation, evolution, and resilience of plant species to environmental changes and emerging threats such as pests and diseases. Assessing genetic diversity provides valuable insights into the number of genotypes, the population structure, and breeding potential. This information facilitates informed decision‐making in crop improvement programs and may contribute to reducing conservation costs [9–11].

Despite its recognized importance, information on the genetic diversity of *O. americanum* in Burkina Faso remains limited. To effectively assess such genetic diversity, reliable and informative molecular tools are required. simple sequence repeat (SSR) markers, also known as microsatellites, are widely used molecular tools for assessing genetic diversity due to their high polymorphism, reproducibility, and codominant inheritance [12–16]. These markers have been successfully applied in various plant species to elucidate population structure, genetic relationships, and evolutionary dynamics [17, 18].

In this study, we aimed to estimate the genetic diversity of *O. americanum* germplasm from Burkina Faso using SSR markers. The specific objectives were to determine the level and organization of genetic diversity and to identify SSR markers suitable for studying genetic diversity in this species.

## 2. Materials and Methods

### 2.1. Plant Material

**FIGURE 1:**
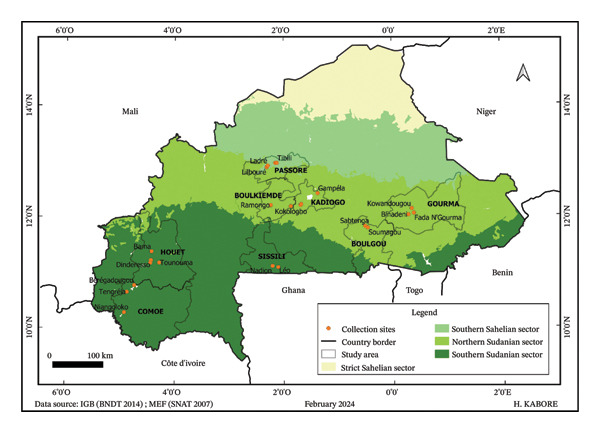
Map of Burkina Faso showing phytogeographic sectors and accession collection sites.

A total of 79 accessions of hoary basil (*O. americanum*) were selected from the gene bank of Joseph KI‐ZERBO University, covering a broad ecological and climatic gradient across Burkina Faso. These accessions originated from eight provinces, representing three of the four phytogeographic sectors of the country (Figure 1). The fourth sector, the strict Sahelian sector, could not be included due to access limitations caused by insecurity, which constrained the collection of *O. americanum* populations.

Sub‐Sahelian Sector: Twenty accessions were obtained from the province of Passoré. This sector receives an average annual rainfall ranging from 500 to 700 mm, with a rainy season lasting three to five months. The main types of vegetation are tiger bushes and thickets [19].

North Sudanian Sector: Thirty accessions were collected from four provinces: Gourma (8 accessions), Kadiogo (10 accessions), Boulkiemdé (8 accessions), and Boulgou (4 accessions). In this sector, the average annual rainfall varies from 700 to 900 mm, with a rainy season lasting five to six months. The landscape is predominantly characterized by savannah vegetation [19].

South Sudanian Sector: Twenty‐nine accessions were gathered from three provinces: Houet (7 accessions), Comoé (15 accessions), and Sissili (7 accessions) (Table 1). This sector experiences an average annual rainfall ranging from 900 to 1100 mm, with a rainy season lasting between six and seven months. The predominant vegetation consists of wooded and tree savannahs, often associated with the presence of waterways [19].

**TABLE 1:** Origin of *O. americanum* accessions.

Phytogeographic sectors	Provinces	Number of accessions	Code of accessions
South Sudanian	Comoé	7	BB4, B45, B06, B40, B7Z1, B50, B44
	Houet	15	BB8, B52, B53, BB1, BZ1, BB7, B51, BB5, B42, B41, BZ4, B4A, BZ2, BZ3, B47
	Sissili	7	BB11, BC1, B112, BB9, BA4, BB12, BA11

North Sudanian	Gourma	8	BB10, B49, BB13, BA1, BB3, BA3, BA5, BA8.
	Kadiogo	10	BC6, B10, B30, B064, B48, B04, B57, B107, B43, B46
	Boulkiemdé	8	24Z, B03, B07, B106, B02, B55, B05, B58
	Boulgou	4	BC2, BC3, BC4, BC5

Sub‐Sahelian	Passoré	20	B09, BA6, B72, B100, BA7, BA9, B08, B102, B104, B103, B59, B54, B60, BA10, BB6, BB2, B56, B01, B531, B105

### 2.2. DNA Extraction

DNA was extracted from fresh leaves of hoary basil accessions using the DNA extraction protocol [20], which was subsequently updated [21]. A sample of 0.2 g of fresh young leaves were ground in 1250 μL of Tris–EDTA–sorbitol buffer (Tris = 100 mM, EDTA = 10 mM, sorbitol = 1 M). The ground samples were then centrifuged at 10,000 rpm for 10 min at 4°C. After the centrifugation, the supernatant was discarded, and 750 μL of prewarmed MATAB (Mixed Alkyl Trimethyl Ammonium Bromide) buffer at 65°C was added to the tubes. The tube contents were mixed by inversion and incubated at 65°C for 2 h and 30 min. After incubation, 750 μL of chloroform isoamyl alcohol (24:1) was added to each sample. The tubes were inverted and then centrifuged at 10,000 rpm for 10 min at 4°C. The aqueous phase was carefully transferred to new 1.5‐mL Eppendorf tubes. Subsequently, 750 μL of fresh 100% isopropanol (4°C) was added to each tube to precipitate the DNA. The supernatant was removed after centrifugation at 10,000 rpm for 10 min at 4°C. The DNA pellet was rinsed once with 500 μL of 70% ethanol for 2 min, air‐dried, and then dissolved in 100 μL of high salt Tris–EDTA buffer before being stored at −20°C. The DNA concentration was measured using a Nanodrop 1000 UV–Vis spectrophotometer, and its purity was assessed by analyzing the optical density at 260 nm and the ratio of optical densities at 260–280 nm.

### 2.3. SSR Marker Genotyping

#### 2.3.1. SSR Markers and Polymerase Chain Reaction (PCR)

A total of 13 microsatellite primers were used to genotype 79 accessions of *O. americanum* (Table 2). These markers were originally developed for *Solenostemon rotundifolius*, targeting di‐, tri‐, tetra‐, penta‐, and hexanucleotide motifs identified as microsatellite regions. Although developed for another species, their use in *O. americanum* is justified by the high conservation of SSR flanking sequences within the *Lamiaceae* family, which allows successful cross‐species amplification when species‐specific markers are not available. Cross‐species transferability of SSR markers has been demonstrated within the genus *Ocimum*, where EST‐derived SSR markers developed for one species have been successfully applied to related taxa [17, 22].

**TABLE 2:** Characteristics of microsatellite (SSR) primers used for the study of genetic diversity in *O. americanum*.

Primers	Motifs	No. rep.	Forward	Reverse
SSRSR04	AG	10	TTA​AGC​AGT​CCA​TGG​CGT​CG	GTG​ATG​TGG​GCC​TAG​GCT​AC
SSRSR05	AC	11	GTGAGGCCGGGTGGATTC	AGC​AAT​CGG​AGA​AGA​CAC​GT
SSRSR17	AAAT	5	TCA​TGC​CTC​CAC​CAA​CAT​GA	GGC​ATG​ATG​ATT​CAA​TCA​CCC​T
SSRSR22	AG	23	AAG​TGG​TGG​ACA​GAG​GCA​GG	ACT​CGT​AGC​CTG​CAA​CAA​CA
SSRSR23	ACT	6	TGG​AGG​CGA​AGA​TGT​CAA​GG	TGG​CCG​CCT​TTG​ATT​AAA​CC
SSRSR26	CCG	7	AAG​GTT​GCT​TGG​TGA​GAG​GG	TCG​CGA​AAG​GCA​GGA​GTA​AA
SSRSR27	AATG	6	TGC​TGC​GAT​ACT​TGA​GCT​GA	TCA​TCG​ATA​TGC​ATA​ACC​ATC​CCT
SSRSR33	CG	5	GGTCGTCTGGGTGTGAGC	CAT​ATT​GTG​CAA​AGA​TGA​GAG​CA
SSRSR36	ACAT	5	GCT​TTC​TCA​TTA​GCA​GCC​ACC	AGC​TAT​GAA​TGA​GAT​CAA​TGC​AGG
SSRSR38	AGAGGG	5	TGG​GAG​CTT​GAT​CTA​CGA​AGC	TGT​GTT​GCT​CAA​CCG​GTT​CT
SSRSR44	CG	6	TAC​ATC​AGA​TGC​GGC​TGT​GG	GTC​CTG​TCG​CAC​GAG​AAC​AA
SSRSR45	ACAGCC	6	CGA​ATC​GTG​GTG​AGG​AGG​G	TCT​TCG​TGC​CGA​CAA​CAA​GA
SSRSR49	AG	14	ACA​CAA​GTG​AGT​GAT​CTC​CCT​C	GCC​ACA​ACC​ACC​ACA​TTT​CC

PCRs were conducted at the Plant Virology and Biotechnology Laboratory of the Environmental, Agricultural Research, and Training Center in Burkina Faso using a SimpliAmp thermal cycler (Life Technologies Holdings Pte Ltd). The PCR mix was prepared in a final volume of 10 μL, comprising 2.0 μL of HOT FIREPol Blend Master Mix (5x), 0.5 μL of both forward and reverse primers (10 μM each), 2.0 μL of genomic DNA template (40 ng/μL), and 5.0 μL of molecular biology grade water.

PCR amplifications were carried out under the following conditions: initial denaturation at 94°C for 4 min, denaturation at 94°C for 30 s, annealing at 55°C for 1 min, and extension at 72°C for 1 min. This cycle was repeated 36 times, followed by a final extension at 72°C for 5 min. The reactions were then held at 4°C until electrophoresis.

#### 2.3.2. Gel Electrophoresis

Gel electrophoresis was performed following the Kirkhouse Trust Horizontal PAGE protocol. After PCR, the amplified DNA fragments were separated on a 6% polyacrylamide gel. The gel solution was prepared by combining 16.5 mL of acrylamide–bis‐acrylamide (19:1), 1.1 mL of Tris–acetate–EDTA (TAE, 50X), 1.8 mL of ammonium persulfate (10%), and 91.7 μL of tetramethyl ethylenediamine (TEMED) and then brought to a final volume of 110.8 mL with distilled water.

Electrophoresis was conducted in 0.5x TAE running buffer at 200 V for 2 h, using 4.5 μL of the amplified PCR products. A 100‐bp DNA ladder (Solis Biodyne) was used to estimate the molecular weight of the amplified products. Following electrophoresis, the gel was submerged in a 0.5 μg/mL ethidium bromide solution for 10 min. The PCR products were visualized and photographed using a Compact Digimage System, UVDI series (MS major science).

### 2.4. Analysis of Genetic Diversity

The study investigated genetic diversity within the entire collection and within each subpopulation, considering the phytogeographic sectors of the accession collection sites (sub‐Sahelian, North Sudanian, and South Sudanian), as well as the genetic groups resulting from genetic structuring. Various genetic parameters were calculated:

- Major allele frequency (MaF %),
- average number of alleles (Na),
- number of different alleles per locus (At),
- number of effective alleles (Ae),
- Shannon diversity index (I),
- observed heterozygosity (Ho),
- expected heterozygosity (He),
- polymorphism information content (PIC),
- the differentiation index (*G*_*S* *T*_), defined by the following formula:
(1) GST=HT−HSHT=1−HSHT,
 where *H*_*S*_ is the average genetic diversity within subpopulations across all populations and
 
*H*_*T*_ represents the total genetic diversity of the entire set of populations considered as a single population (total diversity), and
- number of migrants per generation (*N*_*m*_) calculated according to the formula of Ref. [23],
(2) Nm=1−GST4GSTn/n−12,
 where *n* indicates the number of populations. The higher the value of *N*_*m*_ exceeds 1, the greater the gene flow.

The data analysis was performed using the program GenAIEx 6.5 (including updates from v6.501).

### 2.5. Analysis of Genetic Structure

A model‐based population structure analysis was performed using STRUCTURE 2.3.4 software package [24] with the admixture model and correlated allele frequencies. The number of subpopulations (*K*) was set at 1–10, with 100 replications per *K* value. Each run consisted of 150,000 burn‐in iterations, followed by 200,000 MCMC (Markov chain Monte Carlo) iterations. The ad hoc statistic Delta *K* was calculated to detect populations using the online program STRUCTURE Selector. This diversity index was tested after 999 permutations. In assigning individuals to groups (subpopulations), an accession was considered to belong to a group if more than 80% of its membership probability was derived from that group.

Additionally, the Discriminant Analysis of Principal Components (DAPC) served as a second method to analyze the population structure. DAPC employs *K*‐means clustering of principal components to assign individuals to groups. The determination of the cluster number is guided by the Bayesian Information Criterion (BIC). DAPC analysis was performed using the *R* package “adegenet” [25] within *R* Studio [26].

Analysis of molecular variance (AMOVA) was conducted using GenIAex 6.5 [27] to estimate population genetic differentiation among and within *O. americanum* accessions.

## 3. Results

### 3.1. Genetic Diversity Parameters

Genetic diversity analysis of the *O. americanum* collection revealed that 69.23% of primers (9/13) were polymorphic, demonstrating the utility of these microsatellite markers for characterizing intraspecific diversity. However, the mean allelic richness (4 alleles/locus) and expected heterozygosity (He = 0.239) indicate relatively low genetic diversity. Dinucleotide repeat markers showed predominant polymorphism compared to tri‐ and tetranucleotide motifs, likely reflecting their higher mutation rates and preferential localization in noncoding regions with reduced selective constraints.

Marker SSRSR22 exhibited the highest diversity values (Ae = 1.811, He = 0.449, I = 0.634), while SSRSR36 was the least informative (Ae = 1.054, He = 0.047), indicating substantial locus‐specific variability in discrimination power. The pronounced heterozygosity excess (observed Ho = 0.54 versus expected He = 0.239), coupled with a strongly negative fixation index (Fis = −3.21), suggests active mechanisms maintaining genetic diversity in *O. americanum* populations (Table 3).

**TABLE 3:** Genetic diversity parameters of *O. americanum*.

	**MaF (%)**	**At**	**Ae**	**I**	**Ho**	**He**	**Fis**	**PIC**

SSRSR4	0.81	2	1.349	0.420	0.00	0.258	1.00	0.255
SSRSR5	0.71	3	1.289	0.291	0.77	0.187	−3.12	0.184
SSRSR22	0.65	4	1.811	0.634	0.64	0.449	0.43	0.443
SSRSR26	0.94	2	1.106	0.200	0.00	0.097	1.00	0.096
SSRSR27	0.43	4	1.392	0.368	0.20	0.238	0.16	0.235
SSRSR33	0.87	4	1.765	0.600	0.68	0.421	−0.62	0.415
SSRSR36	0.92	3	1.054	0.090	1.00	0.047	−20.28	0.047
SSRSR44	0.89	7	1.212	0.246	0.96	0.143	−5.71	0.141
SSRSR49	0.53	7	1.520	0.473	0.61	0.314	−0.94	0.310
Mean	0.75	4	1.389	0.369	0.54	0.239	−3.21	0.236

*Note:* At: number of different alleles per primer; Ae: number of effective alleles; Ho: observed heterozygosity; He: expected heterozygosity; I: Shannon’s diversity index; Fis: fixation index.

Abbreviations: MaF, major allele frequency; PIC, polymorphism information content.

### 3.2. Genetic Diversity of *O. americanum* Based on the Phytogeographic Sectors of Origin

Genetic diversity analysis of *O. americanum* accessions reveals a significant differentiation among phytogeographic sectors as determined by AMOVA (*p* < 0.05). Accessions from the South Sudanian sector consistently exhibited lower genetic diversity, as reflected by low values for the number of alleles (A), effective number of alleles (Ae = 1.333), Shannon’s diversity index (I = 0.325), and expected heterozygosity (He = 0.215). These results suggest a more genetically homogeneous population (Table 4). In the sub‐Sahelian sector, the observed values for the effective number of alleles (Ae = 1.41), Shannon’s diversity index (I = 0.356), and expected heterozygosity (He = 0.247) indicate a moderate level of genetic diversity among the accessions studied.

**TABLE 4:** Genetic diversity parameters of the *O. americanum* collection according to phytogeographic sectors.

Phytogeographic sectors	N	A	Ae	I	Ho	He	Gst	Nm
South Sudanian	29.000	3.300	1.333	0.325	0.48	0.215	0.090	1.120
North Sudanian	30.000	3.700	1.443	0.398	0.52	0.271		
Sub‐Sahelian	20.000	3.400	1.410	0.356	0.47	0.247		

*Note:* N: number of accessions; A: mean number of alleles; Ae: number of effective alleles; He: expected heterozygosity; I: Shannon’s diversity index; Gst: differentiation index; and Nm: number of migrants.

In contrast, accessions from the North Sudanian sector exhibited higher genetic variability (Ae = 1.443, I = 0.398, He = 0.271), indicating a richer and more diverse gene pool.

The estimated gene flow (Nm = 1.12) suggests a moderate level of genetic exchange between the phytogeographic sectors, which may reflect some degree of connectivity among them.

The results of the AMOVA based on the method of Excoffier et al. (1992) revealed significant differences in the mean values of molecular variability indices (*p* = 0.004) for the SSR markers across the phytogeographic sectors (Table 5). The minimum Nei distance between accessions from the South Sudanian and North Sudanian sectors is 0.022. The differentiation index (ΦPT = 0.066) between accessions from these two sectors is significant at the 5% level. However, the differentiation indices between accessions from these two sectors and those from the sub‐Sahelian sector are not significant and the minimum Nei distances are low (0.005 and 0.009) (Table 6).

**TABLE 5:** Results of the analysis of molecular variance (AMOVA) for SSR markers among *O. americanum* accessions from the three phytogeographic sectors studied.

Source	Df	SS	MS	Est. var.	*D* (%)
Among phytogeographic sectors	2	19.400	9.700	0.195	4^∗^
Within phytogeographic sectors	76	352.878	4.643	4.643	96
Total	78	372.278		4.838	100

	***Φ*PT value**	**p** **value**			

ΦPT	0.040	0.004			

*Note: D* (%): distribution of total variance.

Abbreviations: df, degrees of freedom; Est. var., estimated variance; MS, mean square; and SS, sum of squares.

^∗^Significant at the 5% level.

**TABLE 6:** Values of the genetic differentiation index (ΦPT) and the minimum Nei distance among accessions of *O. americanum* from different phytogeographic sectors.

	South Sudanian	North Sudanian	Sub‐Sahelian
South Sudanian	0	0.022	0.005
North Sudanian	0.066^∗^	0	0.009
Sub‐Sahelian	0.018 ns	0.024 ns	0

*Note:* The values of the genetic differentiation index and the minimum Nei distance are located, respectively, in the lower triangle and the upper triangle of the table.

^∗^Significant at the 5% threshold.

### 3.3. Population Structure of *O. americanum*

The population structure analysis of *O. americanum* accessions revealed an optimal number of groups with *K* = 2 (Figure 2(a)). These results allow the collection to be organized into two primary groups, each with individual membership coefficients (*Q*) of 80% or higher (Figure 2(b)). The first group comprises 31 accessions, representing accessions from the sub‐Sahelian (10 accessions), North Sudanian (12 accessions), and south Sudanian (9 accessions) phytogeographic sectors. The second group included 6 from the sub‐Sahelian sector, 7 from the North Sudanian sector, and 9 from the South Sudanian sector. In contrast, the remaining 26 accessions are categorized as a mixed group, positioned as intermediate between the identified primary groups. This group consists of 11 accessions from the South Sudanian sector, 11 from the North Sudanian sector, and 4 accessions from the sub‐Sahelian sector.

FIGURE 2Population structure of the 79 accessions of *O. americanum*. (a) Delta *K* graph with its modal value indicating a true *K* of two groups (*K* = 2). (b) Colors represent the two groups based on a membership probability ≥ 80%.

**Figure:**
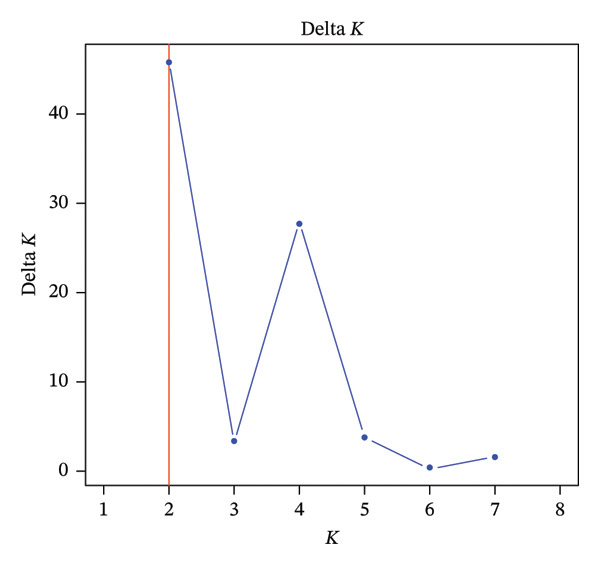
(a)

**Figure:**
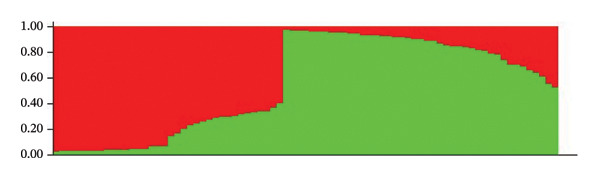
(b)

The DAPC yielded a minimum value for the BIC of six (Figure 3(a)). This DAPC analysis thus identified six genetic groups of *O. americanum* accessions (Figure 3(b)), with a membership probability of 100% for all except accession B40, which had a membership probability of 80% (Figure 3(c)). Each group includes accessions from all three phytogeographic sectors, except for Group 2.

FIGURE 3Results of the discriminant analysis of principal components (DAPC). (a) Curve showing the evolution of the Bayesian information criterion (BIC), with a minimum value of 6 indicating the optimal number of distinct genetic groups in the collection of *O. americanum* accessions. (b) Projection of *O. americanum* accessions onto the plane formed by the first two axes of the discriminant analysis of principal components (DAPC). (c) Membership probabilities of *O. americanum* accessions to the genetic groups highlighted by the DAPC.

**Figure:**
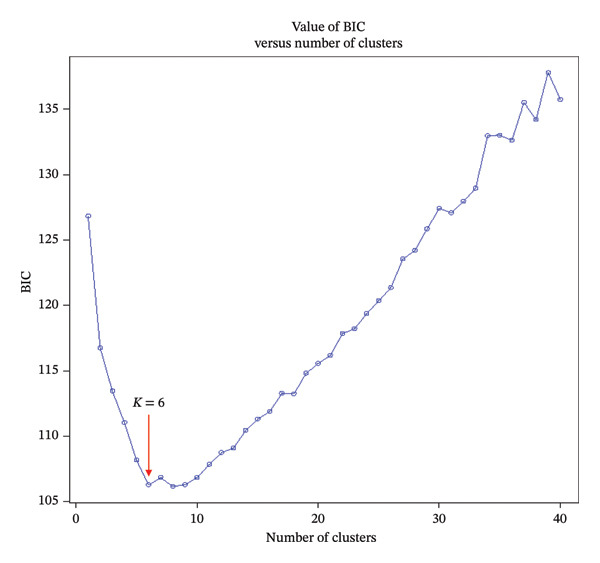
(a)

**Figure:**
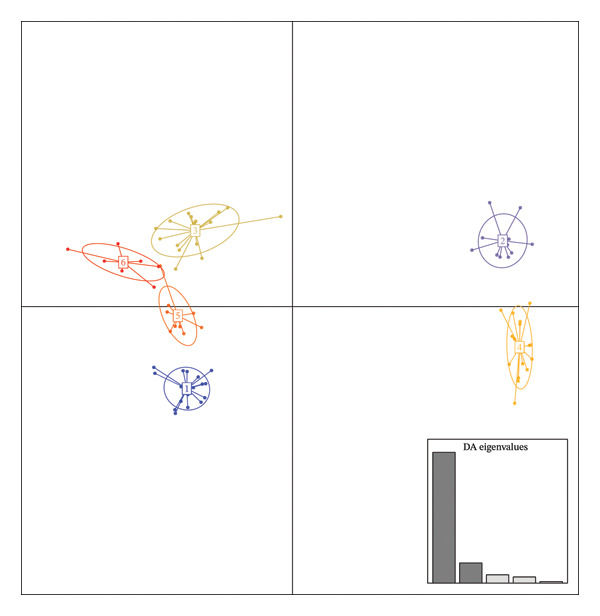
(b)

**Figure:**
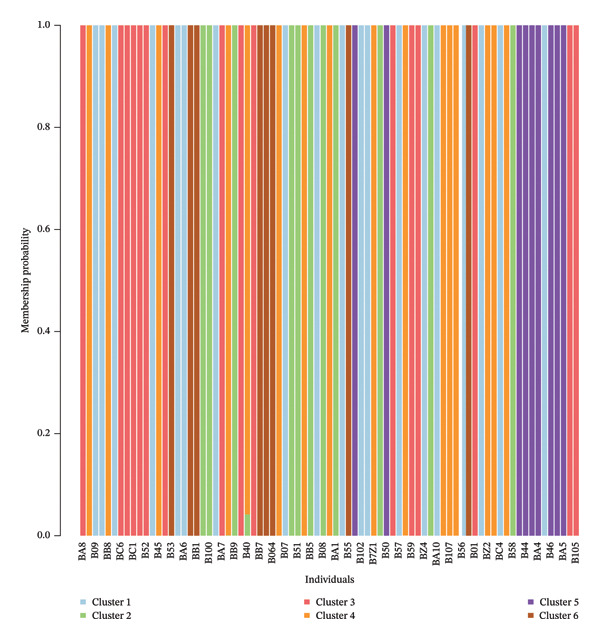
(c)

#### 3.3.1. Characterization of Genetic Groups

The AMOVA revealed significant differences (43.4%) between the two distinct genetic groups identified using the STRUCTURE software. In the AMOVA conducted on the molecular data from the groups obtained by DAPC, the genetic variance among the six subpopulations is low, with a value of 6.7% (Table 7).

**TABLE 7:** Results of the analysis of molecular variance (AMOVA) for the groups of *O. americanum* accessions identified using STRUCTURE and DAPC.

Method	Source	Df	SS	MS	Est. var.	%
Model‐based (STRUCTURE)	Among pops	1	69.991	69.991	2.588	43.4^∗^
	Within pops	51	172.263	3.378	3.378	56.6
	Total	52	242.264		5.996	100

Model‐based (DAPC)	Among pops	5	43.435	8.687	0.324	6.70^∗^
	Within pops	73	328.843	4.505	4.505	93.3
	Total	78	372.278		4.829	100

*Note: D* (%): distribution of total variance.

Abbreviations: df, degrees of freedom; Est. var., estimated variance; MS, mean square; and SS, sum of squares.

^∗^Significant at the 5% threshold.

The two populations identified by STRUCTURE exhibit a significant differentiation index at the 5% threshold, with a minimum Nei distance of 0.018 (Table 8). Regarding the groups identified by DAPC, significant differences were found between Group 6 and the other five groups. Additionally, significant differentiation indices were observed between Group 1 and Groups 4 and 5 (Table 9). The greatest Nei distance was observed between Subpopulations 2 and 6 (*D* = 0.086), while the smallest distance (*D* = 0.004) is found between Subpopulations 2 and 3.

**TABLE 8:** Values of the genetic differentiation index (ΦPT) and the minimum Nei distance between accessions of the two genetic groups revealed using the STRUCTURE software.

	Group 1	Group 2
Group 1	0	0.018
Group 2	0.434^∗^	0

*Note:* The values of the genetic differentiation index and the minimum Nei distance are located, respectively, in the lower triangle and the upper triangle of the table.

^∗^Significant at the 5% level.

**TABLE 9:** Values of the genetic differentiation index (ΦPT) and the minimum Nei distance between accessions of the six genetic groups identified by DAPC.

	Group 1	Group 2	Group 3	Group 4	Group 5	Group 6
Group 1	0	0.021	0.011	0.019	0.027	0.081
Group 2	0.050	0	0.004	0.022	0.019	0.086
Group 3	0.028	0.008	0	0	0	0.060
Group 4	0.056^∗^	0.064	0.002	0	0	0.052
Group 5	0.078^∗^	0.059	0	0.000	0	0.023
Group 6	0.194^∗^	0.209^∗^	0.159^∗^	0.161^∗^	0.096^∗^	0

*Note:* The values of the genetic differentiation index and the minimum Nei distance are located, respectively, in the lower triangle and the upper triangle of the table.

^∗^Significant at the 5% threshold.

## 4. Discussion

Several SSR markers identified in the genome of *S. rotundifolius*, a tuberous herbaceous plant belonging to the *Lamiaceae* family, were successfully amplified in the genome of *O. americanum*. Indeed, 69.23% of these markers revealed polymorphism within the collection of 79 accessions studied. These observations indicate the potential use of SSR markers from one species to study the diversity of another species [28, 29]. The observed polymorphism is consistent with the results reported by several authors who have demonstrated the high capacity of SSR markers to detect genetic polymorphism in plants, as well as their usefulness in gene tagging and genetic mapping [20, 30, 31].

For all loci, the number of alleles per individual varied between 1 and 4. This result aligns with the reported tetraploidy of *O. americanum* [5]. Low PIC values (below 0.50), ranging from 0.047 to 0.443, were observed for all loci, suggesting that the markers used were not very informative. Markers developed for *Vigna unguiculata* (cowpea), a cultivated species of the Fabaceae family, and used to assess the genetic diversity of *Senna obtusifolia*, a wild species belonging to the same family, also revealed PIC values below 0.5 [32]. However, the SSR markers from *S. rotundifolius* can be effectively utilized for the study of diversity in *O. americanum* in the absence of species‐specific markers. According to previous studies [33, 34], even when PIC values are below 0.5, these markers can still provide reliable and informative data for assessing genetic diversity within species.

The excess of observed heterozygosity (Ho = 0.54) relative to expected heterozygosity (He = 0.239) indicates recent genetic admixture between previously distinct subpopulations. This deviation from Hardy–Weinberg equilibrium (HWE) predictions demonstrates that these natural populations have been influenced by human activity, suggesting that *O. americanum* was subject to selection due to its ethnobotanical significance in West African traditional medicine [4]. The concurrent low Shannon diversity index (I = 0.369) with heterozygosity excess reveals that these populations are structured around specific adaptive heterozygous combinations rather than resulting from widespread random mating, indicating potential selective sweeps or domestication‐related population bottlenecks.

This low level of diversity was also observed in accessions of the genus *Ocimum* using AFLP markers [35]. Previous studies have also reported a low level of genetic diversity in wild plants using SSR markers [33]. This low genetic diversity in wild species could be attributed to anthropogenic activities and climate change, which reduce the geographic distribution of species and increase the risk of loss of their genetic diversity [36].

The genetic indices for accessions collected from the north Sudanian phytogeographic sector were higher than those for accessions collected from other phytogeographic sectors. These results may be explained by differences in environmental and climatic factors in the collection areas. The species *O*. *americanum*, being native to Burkina Faso, would be subject to various climatic phenomena and environmental factors, potentially promoting genetic differentiation due to natural selection. The significant genetic differences observed between accessions from the North Sudanian and South Sudanian sectors may result from the maintenance of these two subpopulations in different evolutionary environments without genetic exchange. This genetic differentiation could therefore be influenced by climatic variations and anthropogenic pressures [37].

In contrast, the low genetic differences observed between accessions from the sub‐Sahelian and north Sudanian sectors, as well as between those from the sub‐Sahelian and south Sudanian sectors, suggest a greater genetic similarity between the populations of the sub‐Sahelian and Sudanian sectors. This similarity is reinforced by an average number of migrants (Nm) of 1.12 per generation, indicating gene flow between these populations, which contributes to their genetic homogeneity [17]. This may indicate a greater connectivity between these regions, possibly facilitated by agricultural migrations. Indeed, populations in arid zones, facing challenges of drought, food security, and arable land, tend to converge toward wetland areas [38]. *O*. *americanum* is utilized as a biopesticide in seed conservation; thus, seed exchanges would also facilitate the dissemination of *O. americanum* genetic material, creating genetic proximity between populations from dry and wet zones. Additionally, transhumance could explain the observed genetic proximity [39]. Transhumance is an ancient practice involving the periodic movement of livestock over long distances between seasonal pastures. Animals used in transhumance may inadvertently transport *O*. *americanum* seeds in their feces, contributing to the plant’s long‐distance dispersal. This seed propagation would facilitate genetic exchanges between populations, thus contributing to the genetic similarity observed among accessions of *O*. *americanum* in the sub‐Sahelian and north Sudanian phytogeographic sectors, as well as between accessions from the sub‐Sahelian and south Sudanian sectors.

The analysis of the collection of *O. americanum* accessions using STRUCTURE software revealed two distinct genetic groups, along with a third group comprising 32.91% of the 79 accessions studied, characterized by an intermediate genetic composition. The high value of the differentiation index (ΦPT) between the two genetic groups suggests a strong genetic separation. This may indicate that each group possesses specific adaptations to different environmental or ecological conditions, thereby enhancing their ability to survive and thrive in diverse habitats.

Despite the genetic separation between the groups, a moderate Nei distance implies that there is still some genetic similarity among individuals within each group. However, sufficient genetic diversity is maintained within each population to respond to selection pressures and environmental changes. The intermediate nature of the admixed group between the first two groups may be explained by hybridization. According to a study [40], hybridization is particularly common among species in the *Ocimum* genus. These results indicate that natural crossings have preserved a level of genetic diversity in *O*. *americanum*. The admixed group or hybrid population may have arisen from hybridization between individuals from the previously isolated or ancestral populations, thus playing a role in the diversification and adaptation of these populations.

The DAPC conducted on the 79 basil accessions categorized them into six clusters with an 80% individual assignment probability. This discrepancy with the results obtained using the STRUCTURE approach can be attributed to the fundamental differences between these methods. DAPC, which employs a multivariate approach, is often used for species with less pronounced genetic variation or less distinct genetic structures. In contrast, STRUCTURE, based on the HWE model, is particularly effective at identifying genetic groups with strong genetic differentiation. The results show that DAPC revealed only 6.7% of the molecular variance between the formed groups, whereas STRUCTURE identified 43.4% of the variance between the two genetic groups. This suggests that STRUCTURE may be more suitable for studying the structure of *O. americanum* as it better captures groups with significant genetic divergence. This conclusion contrasts with the findings of some authors [16, 41], which indicated that DAPC is more effective for analyzing the structuring in cassava. Cassava, which reproduces primarily through vegetative propagation, exhibits more homogeneous genetic variation among individuals, which may make DAPC more appropriate for detecting structuring in such plants.

## 5. Conclusion

The study uncovered genetic diversity within *O*. *americanum* collection in Burkina Faso with nine out of 13 nuclear microsatellite markers exhibiting polymorphism. These markers serve as valuable tools for assessing genetic diversity in *O. americanum*.

Despite significant variations observed between accessions from the sub‐Sahelian and South Sudanian sectors as well as between accessions from Groups 1 and 2 and Groups 1 and 3 as defined by the structure, the study indicates minor variations between accessions from the sub‐Sahelian and North Sudanian phytogeographic sectors as well as between accessions from the North Sudanian and South Sudanian sectors. The overall population structure, irrespective of the collection areas, suggests potential interhybridization among accessions. These findings could be instrumental in establishing a breeding program for basil in Burkina Faso. Cytological studies can provide crucial information for the conservation and improvement of *O. americanum*, by helping to clarify its ploidy, evaluate its genetic diversity, identify important characteristics, and develop conservation methods.

## Author Contributions

Conceptualization: Hervé Kaboré and Kiswendsida Romaric Nanema; validation: Hervé Kaboré and Kiswendsida Romaric Nanema; formal analysis: Hervé Kaboré, Monique Soro, and Kiswendsida Romaric Nanema; investigation: Hervé Kaboré; writing original draft: Hervé Kaboré; writing, review, and editing: Hervé Kaboré, Monique Soro, Kiswendsida Romaric Nanema, Ezechiel Bionimian Tibiri, and Fidèle Tiendrebéogo.

## Funding

The authors did not receive any funding for this study.

## Ethics Statement

All *Ocimum americanum* accessions used in this study were obtained from the gene bank of Joseph KI‐ZERBO University. The original collection was carried out with the approval of local authorities and in compliance with national regulations on access to genetic resources and benefit sharing (Nagoya Protocol). All plant materials were ethically sourced and handled in accordance with institutional and national guidelines for the use of plant genetic resources in research.

## Conflicts of Interest

The authors declare no conflicts of interest.

## Data Availability

The data supporting the findings of this study can be obtained from the corresponding author upon request.
